# Supplementary material for: How fast-and-frugal trees can inform diagnostic and intervention decisions for enhancing elite athlete performance
Source: PLoS One. 2025 Aug 18;20(8):e0329395. doi: 10.1371/journal.pone.0329395 (PMC12360579; doi:10.1371/journal.pone.0329395)
Supplement: S1 File — No values indicate diagnostics were not applied in this discipline, Exclusion criteria include high missing values (°), “first step” interventions (*); a = only female athletes, AR/IR = external/internal rotation, CMJ = countermovement jump, d2-R = d2-Test revised version (visual selective attention), DJ = drop jump, D/ND = dominant/ nondominant side, HAST = Handball Agility-Specific Test, KtW = Knee to Wall, KV = strength ratios, rel. = relative, RJ = Repeated Jumps performance decrement, RSI = reactive strength index, Schd = shoulder diagnostics, YBT = Y-Balance Test, ZVT = Zahlenverbindungstest (information-processing speed). (DOCX) [file pone.0329395.s001.docx]

| Variables | Overall (*N* = 466) | | |  | Volleyball FFTree  (*n* = 53) | | |  | Trampoline FFTree  (*n* = 27) | | |
| --- | --- | --- | --- | --- | --- | --- | --- | --- | --- | --- | --- |
|  | *M* |  | *SD* |  | *M* |  | *SD* |  | *M* |  | *SD* |
| Burnout | 2.21 | ± | 0.53 |  | 2.11 | ± | 0.46 |  | 2.42 | ± | 0.60 |
| Social support | 4.50 | ± | 0.46 |  | 4.57 | ± | 0.50 |  | 4.27 | ± | 0.63 |
| Group cohesion | 3.83 | ± | 0.54 |  | 3.96 | ± | 0.38 |  | 3.89 | ± | 0.54 |
| Hedonic balance | 1.13 | ± | 0.78 |  | 1.25 | ± | 0.65 |  | 0.95 | ± | 0.78 |
| Crit. life events | 1.40 | ± | 1.06 |  | 1.22 | ± | 0.95 |  | 1.44 | ± | 1.01 |
| General life satisfaction | 7.15 | ± | 1.51 |  | 7.43 | ± | 1.32 |  | 6.64 | ± | 1.50 |
| ZVT | 108.49 | ± | 10.12 |  | 104.62 | ± | 8.18 |  | 110.85 | ± | 10.73 |
| d2-R | 104.15 | ± | 9.14 |  | 107.63 | ± | 10.37 |  | 103.22 | ± | 9.50 |
| Shannon Index* | 6.02 | ± | 0.57 |  | 6.14 | ± | 0.46 |  | 6.12 | ± | 0.55 |
| Rel. caloric intake (%)* | 71.44 | ± | 20.83 |  | 78.00 | ± | 20.28 |  | 53.50 | ± | 17.43 |
| Vertec (cm)° | 69.57 | ± | 13.05 |  | 69.81 | ± | 11.86 |  |  |  |  |
| CMJ (cm) | 37.02 | ± | 7.76 |  | 37.99 | ± | 8.29 |  | 31.39 | ± | 6.33 |
| DJ RSI (m/s) | 1.40 | ± | 0.37 |  | 1.47 | ± | 0.40 |  |  |  |  |
| 10 m Sprint (s) | 1.86 | ± | 0.14 |  | 1.89 | ± | 0.15 |  |  |  |  |
| HAST (s)° | 7.59 | ± | 0.63 |  | 7.60 | ± | 0.66 |  |  |  |  |
| Medicine ball (m)° | 9.44 | ± | 1.98 |  | 9.93 | ± | 2.34 |  |  |  |  |
| Tapping (Hz) | 11.42 | ± | 1.35 |  | 11.48 | ± | 1.02 |  | 10.61 | ± | 1.33 |
| Motor cost (Hz) | 1.49 | ± | 1.10 |  | 1.39 | ± | 1.03 |  | 2.40 | ± | 1.59 |
| Motor Inhibition (ms) | 234.91 | ± | 42.43 |  | 234.59 | ± | 34.56 |  | 238.59 | ± | 34.99 |
| YBT | 98.94 | ± | 7.37 |  | 97.65 | ± | 6.51 |  | 101.49 | ± | 7.31 |
| KtW (cm) | 13.35 | ± | 3.25 |  | 13.71 | ± | 3.29 |  | 11.44 | ± | 2.95 |
| Rel. Grip strength (N/kg) | 3.33 | ± | 0.65 |  | 3.18 | ± | 0.61 |  | 3.55 | ± | 0.70 |
| RJ° | -9.78 | ± | 4.35 |  |  |  |  |  | -10.69 | ± | 4.49 |
| Schd_KV_Th01_D/ND° | 1.07 | ± | 0.12 |  | 1.06 | ± | 0.12 |  |  |  |  |
| Schd_KV_Th02_D/ND° | 1.03 | ± | 0.13 |  | 1.00 | ± | 0.14 |  |  |  |  |
| Schd_KV_Th08_D/ND° | 1.11 | ± | 0.21 |  | 1.16 | ± | 0.24 |  |  |  |  |
| Schd_KV_Th10_D/ND° | 1.09 | ± | 0.19 |  | 1.10 | ± | 0.18 |  |  |  |  |
| Schd_KV_AR/IR_reT1/T2° | 0.69 | ± | 0.11 |  | 0.66 | ± | 0.11 |  |  |  |  |
| Schd_01_re_rel (N/kg)° | 2.26 | ± | 0.41 |  | 2.32 | ± | 0.45 |  |  |  |  |
| Schd_08_re_rel (N/kg)° | 0.84 | ± | 0.31 |  | 0.80 | ± | 0.27 |  |  |  |  |
| Schd_10_re_rel (N/kg)° | 1.56 | ± | 0.40 |  | 1.56 | ± | 0.40 |  |  |  |  |
| Cycle symptoms^a^° | 2.08 | ± | 1.92 |  | 2.86 | ± | 2.10 |  | 2.07 | ± | 1.39 |
