# Supplementary material for: How fast-and-frugal trees can inform diagnostic and intervention decisions for enhancing elite athlete performance
Source: PLoS One. 2025 Aug 18;20(8):e0329395. doi: 10.1371/journal.pone.0329395 (PMC12360579; doi:10.1371/journal.pone.0329395)
Supplement: S2 File — (DOCX) [file pone.0329395.s002.docx]

A total of 16 variables were analyzed across the entire sample. To verify the factorability of the data, the correlation matrix was inspected on correlations above an absolute value of *r* > .30, and a Bartlett test assessed their significance. Additionally, the Kaiser–Meyer–Olkin (KMO) test of sampling adequacy with values above .60 was required for a functioning PCA (56).

An explorative data analysis via principal component analysis with varimax rotation was conducted. Orthogonal rotation is recommended if PCA results are to be used in further analysis (56). The number of extracted components was determined with eigenvalues above 1 and a visual interpretation of the scree plot. To test for the presence of clustered components, the PCA was analyzed one below and above the Kaiser criterion. In line with Tabachnick and Fidell (56), the component loadings were interpreted as “excellent” with values above .71, as “very good” above .63, as “good” above .55, as “fair” above .45 and as “poor” above .32. Only fair loadings were interpreted (56). Missing values were excluded pairwise.
