# Supplementary material for: How fast-and-frugal trees can inform diagnostic and intervention decisions for enhancing elite athlete performance
Source: PLoS One. 2025 Aug 18;20(8):e0329395. doi: 10.1371/journal.pone.0329395 (PMC12360579; doi:10.1371/journal.pone.0329395)
Supplement: S3 File — Extraction method: Principal component analysis with varimax rotation; CMJ = countermovement jump, DJ = drop jump, KtW = Knee to Wall, rel. = relative, RSI = reactive strength index, YBT = Y-Balance Test. (DOCX) [file pone.0329395.s003.docx]

|  | Components | | | | | |
| --- | --- | --- | --- | --- | --- | --- |
|  | 1 | 2 | 3 | 4 | 5 | 6 |
| Burnout | 0,04 | -0,75 | -0,01 | 0,09 | 0,09 | -0,02 |
| Social support | -0,00 | 0,56 | -0,01 | 0,08 | 0,09 | 0,14 |
| Group cohesion | 0,01 | 0,67 | 0,01 | 0,03 | -0,18 | 0,07 |
| Hedonic balance | -0,03 | 0,76 | 0,03 | -0,01 | 0,10 | -0,10 |
| General life satisfaction | -0,11 | 0,62 | -0,10 | -0,07 | 0,22 | -0,20 |
| Information-processing speed | -0,01 | -0,02 | 0,84 | 0,10 | -0,12 | 0,13 |
| Visual selective attention | -0,01 | -0,02 | 0,81 | -0,04 | 0,15 | -0,21 |
| CMJ | 0,78 | -0,04 | -0,01 | 0,11 | -0,08 | -0,03 |
| DJ RSI | 0,74 | -0,03 | 0,03 | -0,08 | -0,17 | -0,19 |
| 10 m sprint | -0,69 | 0,04 | 0,17 | -0,09 | -0,19 | -0,05 |
| Tapping | 0,63 | -0,04 | 0,13 | 0,06 | 0,29 | 0,16 |
| Motor cost | 0,09 | 0,07 | 0,04 | -0,14 | 0,82 | 0,06 |
| Motor inhibition | -0,03 | 0,01 | -0,06 | -0,01 | 0,05 | 0,93 |
| YBT | 0,34 | -0,01 | 0,09 | 0,69 | -0,15 | 0,01 |
| KtW | -0,14 | -0,02 | -0,01 | 0,86 | 0,03 | -0,01 |
| Rel. grip strength | 0,47 | -0,01 | 0,04 | -0,18 | -0,41 | 0,03 |
