# Supplementary material for: How fast-and-frugal trees can inform diagnostic and intervention decisions for enhancing elite athlete performance
Source: PLoS One. 2025 Aug 18;20(8):e0329395. doi: 10.1371/journal.pone.0329395 (PMC12360579; doi:10.1371/journal.pone.0329395)
Supplement: S4 File — FFTree for trampoline generated for the training dataset with 60% of the subsample via the formula: FFTrees(formula = WKOlymp ~ ZL6_HedBalance + ZKL_SW +. ZGriffk_Re_rel + ZYBAL_Composite_Re + ZMotCost_TapStr + ZInhi_SSRT_Hand, data = HeuristicTreeTrampolin, train.p = 0.6) and FFTree for volleyball generated with the training dataset with 60% of the subsample via the formula: FFTrees(formula = WKOlymp ~ ZL6_HedBalance + ZZVT_SW + ZCMJ_bb + ZYBAL_Composite_Re + ZMotCost_TapStr + ZInhi_SSRT_Hand, data = HeuristicTreeVolleyball_Lib, train.p = 0.6); acc = Accuracy, bacc = balanced accuracy, d2-R = d2-Test revised version (visual selective attention), CMJ = countermovement jump, mcu = mean cues used, pci = percentage cues ignored, ROC = receiver operating characteristic (presenting the performance of all FFTrees from the “fan” based on the bacc with false alarm rate [FAR] on the x and sensitivity on the y axis), sens = sensitivity, spec = specificity, YBT = Y-Balance Test. (PDF) [file pone.0329395.s004.pdf]

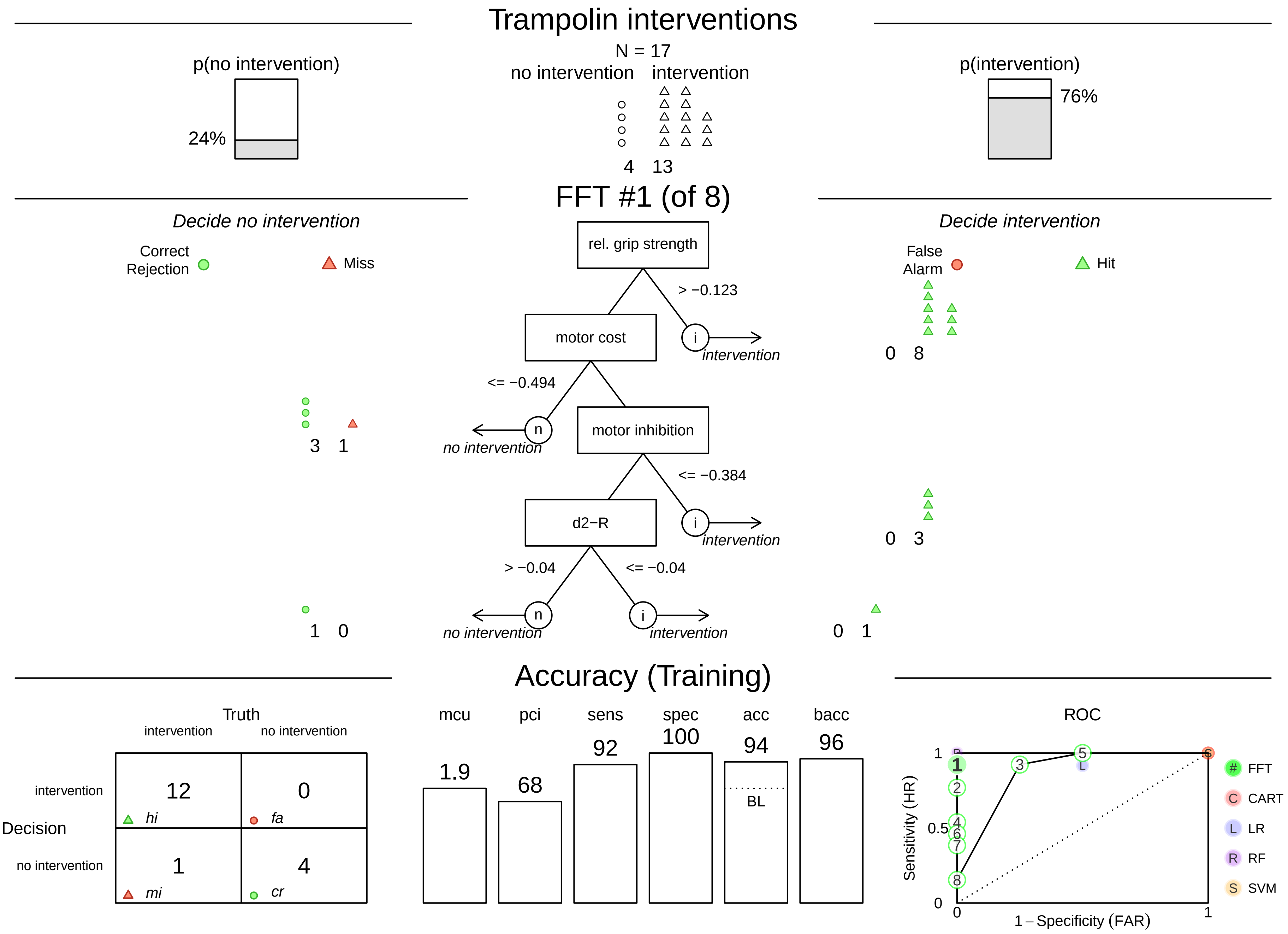
Trampolin interventions
N = 17
p(no intervention)
24%
no intervention
intervention
4
13
p(intervention)
76%
FFT #1 (of 8)
Decide no intervention
Correct Rejection
Miss
rel. grip strength
> −0.123
motor cost
i
intervention
<= −0.494
n
no intervention
motor inhibition
<= −0.384
d2−R
> −0.04
<= −0.04
Decide intervention
False Alarm
Hit
0 8
3 1
0 3
1 0
0 1
Accuracy (Training)
Truth
intervention
no intervention
Decision
12
hi
0
fa
1
mi
4
cr
mcu
1.9
pci
68
sens
92
spec
100
acc
94
BL
bacc
96
ROC
Sensitivity (HR)
1 – Specificity (FAR)
FFT
CART
LR
RF
SVM

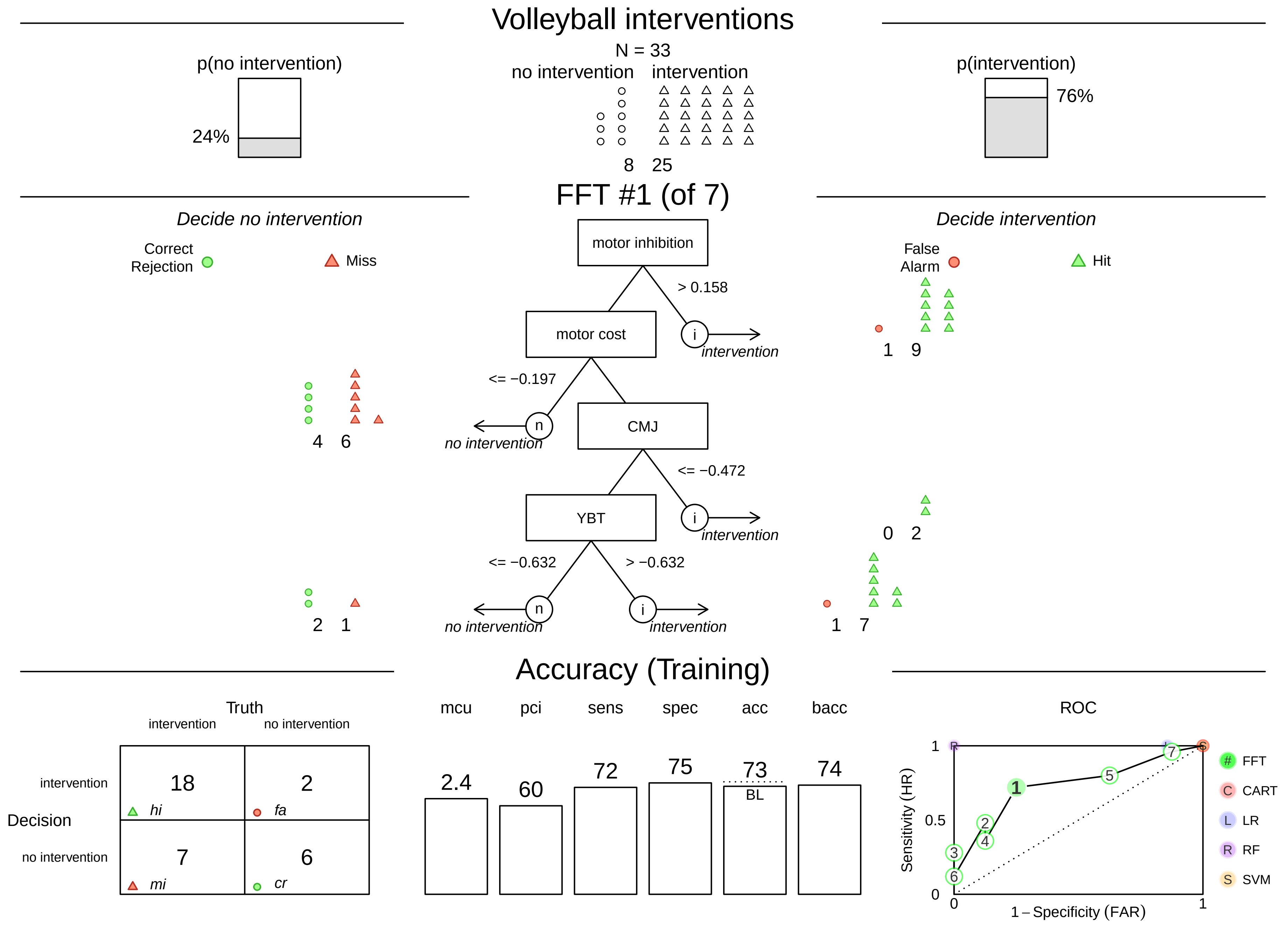

Volleyball interventions
N = 33
p(no intervention)
24%
no intervention
intervention
8
25
p(intervention)
76%
FFT #1 (of 7)
Decide no intervention
Correct Rejection
Miss
motor inhibition
> 0.158
i
intervention
motor cost
<= −0.197
n
no intervention
CMJ
<= −0.472
i
intervention
YBT
<= −0.632
> −0.632
n
no intervention
i
intervention
Decide intervention
False Alarm
Hit
4 6
2 1
1 9
0 2
1 7
Accuracy (Training)
Truth
intervention
no intervention
Decision
intervention
no intervention
18 hi
2 fa
7 mi
6 cr
mcu
pci
sens
spec
acc
bacc
2.4
60
72
75
73
BL
74
ROC
Sensitivity (HR)
1 – Specificity (FAR)
0
0.5
1
0
1
FFT
CART
LR
RF
SVM
