# Supplementary material for: How fast-and-frugal trees can inform diagnostic and intervention decisions for enhancing elite athlete performance
Source: PLoS One. 2025 Aug 18;20(8):e0329395. doi: 10.1371/journal.pone.0329395 (PMC12360579; doi:10.1371/journal.pone.0329395)
Supplement: S5 File — Twenty random generated FFTrees for trampoline as well as for volleyball and their performance are represented for the training and test data as well as mean (M) and standard deviation (SD), used cues are listed in descending order; acc = accuracy, bacc = balanced accuracy, CMJ = countermovement jump, d2-R = d2-Test revised version (visual selective attention), HedBalance = hedonic Balance, Inhibition = motor inhibition, mcu = mean cues used, MotCost = motor cost, pci = percent cues ignored, Sens = sensitivity, Spec = specificity, YBT = Y-Balance Test, ZVT = Zahlenverbindungstest (information-processing speed). (DOCX) [file pone.0329395.s005.docx]

S5.1 Randomly generated FFTrees.

|  | Training | |  |  |  |  | Test |  |  |  |  |  |  |
| --- | --- | --- | --- | --- | --- | --- | --- | --- | --- | --- | --- | --- | --- |
| Tree | acc | bacc | Sens | Spec | mcu | pci | acc | bacc | Sens | Spec | mcu | pci | Cues |
| 1 | 0.94 | 0.96 | 0.92 | 1.00 | 1.94 | 0.68 | 0.70 | 0.44 | 0.88 | 0.00 | 1.30 | 0.78 | Grip strength, YBT, MotCost, Inhibition |
| 2 | 0.77 | 0.85 | 0.69 | 1.00 | 2.06 | 0.66 | 0.80 | 0.88 | 0.75 | 1.00 | 1.90 | 0.68 | Grip strength, MotCost, HedBalance, YBT |
| 3 | 0.88 | 0.92 | 0.85 | 1.00 | 2.12 | 0.65 | 0.80 | 0.88 | 0.75 | 1.00 | 2.30 | 0.62 | Grip strength, MotCost, Inhibition, d2-R |
| 4 | 0.88 | 0.92 | 0.85 | 1.00 | 1.35 | 0.77 | 0.70 | 0.44 | 0.88 | 0.00 | 1.20 | 0.80 | Grip strength, YBT |
| 5 | 0.94 | 0.88 | 1.00 | 0.75 | 1.71 | 0.72 | 0.80 | 0.69 | 0.88 | 0.50 | 2.00 | 0.67 | Inhibition, HedBalance, d2-R |
| 6 | 0.94 | 0.96 | 0.92 | 1.00 | 1.94 | 0.68 | 0.90 | 0.94 | 0.88 | 1.00 | 2.40 | 0.60 | Grip strength, MotCost, Inhibition, d2-R |
| 7 | 0.94 | 0.96 | 0.92 | 1.00 | 1.35 | 0.75 | 0.60 | 0.38 | 0.75 | 0.00 | 1.20 | 0.80 | Grip strength, YBT |
| 8 | 0.94 | 0.96 | 0.92 | 1.00 | 1.76 | 0.71 | 0.80 | 0.50 | 1.00 | 0.00 | 1.40 | 0.77 | MotCost, Inhibition, Grip strength |
| 9 | 1.00 | 1.00 | 1.00 | 1.00 | 1.35 | 0.77 | 0.50 | 0.31 | 0.63 | 0.00 | 1.50 | 0.75 | HedBalance, d2-R |
| 10 | 0.88 | 0.92 | 0.65 | 1.00 | 1.53 | 0.75 | 0.50 | 0.50 | 0.50 | 0.50 | 1.80 | 0.70 | Grip strength, YBT, Inhibition |
| 11 | 1.00 | 1.00 | 1.00 | 1.00 | 2.24 | 0.63 | 0.80 | 0.88 | 0.75 | 1.00 | 2.50 | 0.58 | Grip strength, MotCost, Inhibition, HedBalance |
| 12 | 1.00 | 1.00 | 1.00 | 1.00 | 2.00 | 0.67 | 0.80 | 0.50 | 1.00 | 0.00 | 2.00 | 0.67 | d2-R, HedBalance, Grip strength, Inhibition |
| 13 | 0.94 | 0.96 | 0.92 | 1.00 | 2.00 | 0.67 | 0.90 | 0.94 | 0.88 | 1.00 | 2.60 | 0.57 | Grip strength, MotCost, HedBalance, Inhibition |
| 14 | 0.94 | 0.96 | 0.92 | 1.00 | 1.94 | 0.68 | 0.80 | 0.50 | 1.00 | 0.00 | 1.50 | 0.75 | MotCost, Grip strength, YBT, Inhibition |
| 15 | 0.88 | 0.92 | 0.85 | 1.00 | 1.88 | 0.69 | 0.80 | 0.88 | 0.75 | 1.00 | 2.50 | 0.58 | Grip strength, MotCost, HedBalance, Inhibition |
| 16 | 0.88 | 0.92 | 0.85 | 1.00 | 1.94 | 0.68 | 0.40 | 0.44 | 0.38 | 0.50 | 2.20 | 0.63 | Grip strength, HedBalance, d2-R |
| 17 | 0.88 | 0.92 | 0.85 | 1.00 | 1.71 | 0.72 | 0.70 | 0.63 | 0.75 | 0.50 | 1.40 | 0.77 | Grip strength, YBT, Inhibition, d2-R |
| 18 | 0.71 | 0.81 | 0.62 | 1.00 | 1.71 | 0.72 | 0.69 | 0.75 | 0.50 | 1.00 | 1.80 | 0.70 | Grip strength, MotCost, d2-R, YBT |
| 19 | 0.88 | 0.84 | 0.92 | 0.75 | 1.65 | 0.73 | 0.90 | 0.75 | 1.00 | 0.50 | 1.30 | 0.78 | MotCost, Grip strength, Inhibition |
| 20 | 0.88 | 0.92 | 0.85 | 1.00 | 2.12 | 0.65 | 0.80 | 0.88 | 0.75 | 1.00 | 2.20 | 0.63 | Grip strength, MotCost, Inhibition, HedBalance |
| ***M*** | **0.91** | **0.93** | **0.87** | **0.98** | **1.82** | **0.70** | **0.73** | **0.65** | **0.78** | **0.53** | **1.85** | **0.69** |  |
| ***SD*** | **0.07** | **0.05** | **0.11** | **0.08** | **0.27** | **0.04** | **0.14** | **0.21** | **0.18** | **0.44** | **0.48** | **0.08** |  |

Sport discipline: Trampoline

S5.2

|  | Training | |  |  |  |  | Test |  |  |  |  |  |  |
| --- | --- | --- | --- | --- | --- | --- | --- | --- | --- | --- | --- | --- | --- |
| Tree | acc | bacc | Sens | Spec | mcu | pci | acc | bacc | Sens | Spec | mcu | pci | Cues |
| 1 | 0.82 | 0.80 | 0.84 | 0.75 | 1.97 | 0.67 | 0.65 | 0.41 | 0.81 | 0.00 | 1.65 | 0.72 | CMJ, Inhibition, MotCost, HedBalance |
| 2 | 0.82 | 0.84 | 0.80 | 0.88 | 2.85 | 0.53 | 0.70 | 0.53 | 0.81 | 0.25 | 2.40 | 0.60 | ZVT, YBT, MotCost, HedBalance |
| 3 | 0.76 | 0.80 | 0.72 | 0.88 | 2.61 | 0.57 | 0.55 | 0.34 | 0.69 | 0.00 | 2.35 | 0.61 | CMJ, YBT, ZVT, Inhibition |
| 4 | 0.82 | 0.80 | 0.84 | 0.75 | 2.27 | 0.62 | 0.65 | 0.50 | 0.75 | 0.25 | 2.50 | 0.58 | CMJ, Inhibition, MotCost, HedBalance |
| 5 | 0.70 | 0.80 | 0.60 | 1.00 | 2.39 | 0.60 | 0.40 | 0.63 | 0.25 | 1.00 | 2.60 | 0.57 | MotCost, Inhibition, HedBalance, CMJ |
| 6 | 0.61 | 0.74 | 0.48 | 1.00 | 2.06 | 0.66 | 0.55 | 0.53 | 0.56 | 0.50 | 2.00 | 0.67 | ZVT, MotCost, Inhib, CMJ |
| 7 | 0.73 | 0.78 | 0.68 | 0.88 | 2.61 | 0.57 | 0.50 | 0.50 | 0.50 | 0.50 | 3.10 | 0.48 | MotCost, YBT, CMJ, ZVT |
| 8 | 0.85 | 0.86 | 0.84 | 0.88 | 2.61 | 0.57 | 0.60 | 0.56 | 0.63 | 0.50 | 3.10 | 0.48 | CMJ, YBT, MotCost, Inhibition |
| 9 | 0.85 | 0.91 | 0.81 | 1.00 | 2.96 | 0.51 | 0.58 | 0.55 | 0.60 | 0.50 | 2.92 | 0.51 | YBT, HedBalance, Inhibitor, MotCost |
| 10 | 0.85 | 0.85 | 0.86 | 0.83 | 2.22 | 0.63 | 0.69 | 0.45 | 0.90 | 0.00 | 1.62 | 0.73 | MotCost, ZVT, CMJ, HedBalance |
| 11 | 0.82 | 0.88 | 0.76 | 1.00 | 2.21 | 0.63 | 0.40 | 0.25 | 0.50 | 0.00 | 2.10 | 0.65 | CMJ, Inhibition, HedBalance |
| 12 | 0,73 | 0.78 | 0.68 | 0.88 | 2.55 | 0.58 | 0.70 | 0.63 | 0.75 | 0.50 | 2.55 | 0.58 | HedBalance, CMJ, Inhibition, YBT |
| 13 | 0.85 | 0.73 | 0.96 | 0.50 | 1.82 | 0.70 | 0.55 | 0.34 | 0.69 | 0.00 | 2.25 | 0.62 | HedBalance, Inhibition, CMJ, MotCost |
| 14 | 0.82 | 0.84 | 0.80 | 0.88 | 2.52 | 0.58 | 0.45 | 0.56 | 0.38 | 0.75 | 2.75 | 0.54 | MotCost, ZVT, Inhibition, CMJ |
| 15 | 0.85 | 0.90 | 0.80 | 1.00 | 2.61 | 0.57 | 0.50 | 0.31 | 0.63 | 0.00 | 2.95 | 0.51 | CMJ, YBT, HedBalance, ZVT |
| 16 | 0.67 | 0.78 | 0.56 | 1.00 | 2.70 | 0.55 | 0.75 | 0.75 | 0.75 | 0.75 | 2.50 | 0.58 | MotCost, ZVT, CMJ, HedBalance |
| 17 | 0.82 | 0.84 | 0.80 | 0.88 | 1.97 | 0.67 | 0.55 | 0.34 | 0.69 | 0.00 | 1.85 | 0.69 | Inhibition, MotCost, ZVT |
| 18 | 0.67 | 0.78 | 0.56 | 1.00 | 2.58 | 0.63 | 0.60 | 0.47 | 0.69 | 0.25 | 2.35 | 0.66 | CMJ, ZVT, Inhibition, MotCost |
| 19 | 0.73 | 0.74 | 0.72 | 0.75 | 2.42 | 0.60 | 0.75 | 0.66 | 0.81 | 0.50 | 2.60 | 0.57 | Inhibition, MotCost, CMJ, YBT |
| 20 | 0.76 | 0.80 | 0.72 | 0.88 | 2.33 | 0.61 | 0.40 | 0.44 | 0.38 | 0.50 | 2.55 | 0.58 | CMJ, MotCost, ZVT, Inhibition |
| ***M*** | **0.77** | **0.81** | **0.74** | **0.88** | **2.41** | **0.60** | **0.58** | **0.49** | **0.64** | **0.34** | **2.43** | **0.60** |  |
| ***SD*** | **0.07** | **0.05** | **0.12** | **0.13** | **0.30** | **0.05** | **0.11** | **0.13** | **0.17** | **0.31** | **0.43** | **0.07** |  |

Sport discipline: Volleyball
