# Supplementary material for: How fast-and-frugal trees can inform diagnostic and intervention decisions for enhancing elite athlete performance
Source: PLoS One. 2025 Aug 18;20(8):e0329395. doi: 10.1371/journal.pone.0329395 (PMC12360579; doi:10.1371/journal.pone.0329395)
Supplement: S6 File — Descending prioritization for the multidisciplinary diagnostics by the coaches from DTB and DVV. The menstrual cycle is only considered for female athletes; DTB = Deutscher Turner-Bund, DVV = Deutscher Volleyball-Verband. (DOCX) [file pone.0329395.s006.docx]

| Categories |  | DTB | DVV |
| --- | --- | --- | --- |
| Physiology | Blood | 1 | 3 |
|  | Microbiome/Nutrition | 2 | 4 |
|  | Genetic | 6 | 5 |
| Psychosociology | Chronic sport-related stress | 5 | 8 |
|  | Drop out | 7 | 6 |
|  | Social support | 8 | 9 |
|  | Sport-related dissatisfaction | 4 | 7 |
| Motor | Performance-related test | 3 | 1 |
| Cognition | Concentration, task, information processing | 9 | 2 |
| Menstrual cycle |  | 10 | 4,5 |
