# Supplementary material for: How fast-and-frugal trees can inform diagnostic and intervention decisions for enhancing elite athlete performance
Source: PLoS One. 2025 Aug 18;20(8):e0329395. doi: 10.1371/journal.pone.0329395 (PMC12360579; doi:10.1371/journal.pone.0329395)
Supplement: Attachment — Submitted filename: 17971_FFT.pdf [file pone.0329395.s007.pdf]

Title of Manuscript: Fast-and-frugal trees: A decision tool for elite sports

Manuscript ID: PONE-D-25-17971

Reviewer's report

First, I would like to warmly acknowledge the considerable effort behind this manuscript. The multidisciplinary approach, the ambition to simplify complex decision-making through FFTrees, and the relevance to elite sports settings are evident throughout the work. My comments aim to support the authors in refining the manuscript to meet the high scientific and editorial standards required by PLOS ONE. I have structured the feedback section by section, with explicit suggestions that I hope will be helpful in moving the paper forward.

1. Abstract

Suggestion: The structure could be improved for clarity. Consider explicitly segmenting the abstract into: Objective, Methods, Results, Conclusion.

For example:

Line 6–7: You may consider beginning with a clear objective such as: "The objective of this study was to examine the applicability of fast-and-frugal trees (FFTrees) to inform training interventions in elite athletes."

Clarity of FFTrees: As this might be unfamiliar to some readers, consider briefly defining what FFTrees are in one sentence near the start.

2. Introduction

Objective not clearly stated:

Lines 101-104: The objective appears but is somewhat embedded. You may consider adding a clear and concise sentence like:

"The aim of this study is to develop and evaluate the predictive performance of sport-specific FFTrees to inform personalised training interventions in elite athletes."

Theory clarification:

Lines 31-40: You refer to theoretical models but do not fully explain how these support the rationale for FFTrees. Consider integrating the constructivist or biopsychosocial lens more explicitly to frame why FFTrees are an appropriate tool for this context.

Suggestion for structure: You might consider adding subheadings or clearly defined paragraphs separating:

- ✓ Context and problem (elite sports complexity)
- ✓ Existing gaps in intervention selection
- ✓ Introduction to FFTrees as a methodological response

3. Methods

Cue Selection Explanation:

Lines 210-225: The PCA is well described but consider clarifying why each variable was selected as a cue. For example, in line 299-301, you could briefly mention the rationale for replacing information-processing speed with visual selective attention in trampoline.

Clarify Dichotomisation Criteria:

Lines 234-245: Please make the thresholding process more transparent. For instance:

- ✓ How was the "75th percentile" decided?
- ✓ Were sex and age considered in the dichotomisation?

Reproducibility:

Lines 272-273: You may consider adding the R script or relevant code as supplementary material or sharing a GitHub link, in line with open science practices.

4. Results

Table 3.1 (line 293): It would be helpful to add a brief sentence interpreting the practical relevance of each component, what does "motor cost" imply, for instance?

Figures 3.1 and 3.2:

The decision rules could be better explained in-text. For example, in line 327-328, you might add:

- ✓ "This threshold suggests that athletes with grip strength z-scores > -0.12 are classified as needing intervention, reflecting possible strength deficiencies."

Missing data handling:

Line 256-258: Please state how many cases were excluded due to missing data, or clarify if imputation was considered.

5. Discussion

Theoretical anchoring:

Lines 382-390: While you reference the multidisciplinary and heuristic value of FFTrees, it would strengthen the paper to briefly connect to models such as constructivist decision-making or ecological dynamics, especially since you touch on bounded rationality. This would deepen the theoretical contribution.

Practical implications:

Lines 445-455: Consider adding an example of how a coach could use a FFTree in a training context. For example:

"A coach reviewing FFTree output could prioritise strength-based interventions if the tree identifies grip strength or CMJ as first-level cues."

Limitations:

While limitations are mentioned (Lines 491-494), it would benefit from a clearer subsection or paragraph that includes:

- ✓ Small sample sizes for team sports

- ✓ Model instability across random partitions
- ✓ Generalisation limitations to youth or non-German athletes

6. Conclusion

Line 511 onward: The conclusion is strong but consider softening claims slightly to reflect the proof-of-concept nature of the study. For example:

“These preliminary findings suggest that FFTrees may offer a practical, transparent method for guiding individualised training decisions, though further research with larger samples is recommended.”

7. Language and style

Orthographic errors:

Line 101: “and whether they can be validated…” is incomplete or has a clause error.

Line 249, 304, and several others: Consider reviewing sentence punctuation. Several sentences are long and lack commas, which makes them harder to follow, this may stem from AI-assisted generation.

Recommendation: A thorough proofreading by a native English speaker or professional editor would improve clarity and flow and meet the PLOS ONE criterion for “standard English.”

Final recommendation: major revision

This is a promising and well-structured study with a novel approach, but it would benefit from clearer articulation of objectives, tighter theoretical framing, enhanced methodological transparency, and improvements to writing and formatting.

I encourage the authors to consider the suggestions outlined above, not as criticisms, but as constructive steps toward strengthening their valuable contribution to performance science.
