# Supplementary material for: How fast-and-frugal trees can inform diagnostic and intervention decisions for enhancing elite athlete performance
Source: PLoS One. 2025 Aug 18;20(8):e0329395. doi: 10.1371/journal.pone.0329395 (PMC12360579; doi:10.1371/journal.pone.0329395)
Supplement: Attachment — Submitted filename: Response to Reviewers.docx [file pone.0329395.s008.docx]

Manuscript ID: PONE-D-25-17971

Response to Reviewers

**Dear Editors and Reviewers**, thank you for allowing us to submit a revised draft of our manuscript. We appreciate you and the reviewers for your precious time in reviewing our paper and providing valuable comments. It was your insightful feedback that led to improvements in the renewed version. We fully and thoroughly addressed your concerns. In this file, we elaborated on your comments in a point-to-point manner and presented consecutive changes to the manuscript. We hope that, after careful revisions, the manuscript meets your standards. The authors welcome further constructive feedback if any.

Sincerely

Lena Siebert and coauthors

| **Comments Editors** | **Answer** | **Changes** |
| --- | --- | --- |
| Please ensure that your manuscript meets PLOS ONE's style requirements, including those for file naming. The PLOS ONE style templates can be found at <https://journals.plos.org/plosone/s/file?id=wjVg/PLOSOne_formatting_sample_main_body.pdf> and <https://journals.plos.org/plosone/s/file?id=ba62/PLOSOne_formatting_sample_title_authors_affiliations.pdf> | Thank you for your request. We have revised the style requirements and updated the formatting sentences in accordance with the PLOS ONE formatting guidelines. We hope our manuscript now completely fulfills the guidelines. | We removed bold type from the article title and updated the author information section to clear out unnecessary information and adapt to the PLOS ONE formatting template.  Changed to justified text and added line numbering for the title page as well as the abstract (separate to the main text).  Numbers in Table 3.1 are aligned to the right for improved clarity. |
| Please note that PLOS ONE has specific guidelines on code sharing for submissions in which author-generated code underpins the findings in the manuscript. In these cases, we expect all author-generated code to be made available without restrictions upon publication of the work. Please review our guidelines at <https://journals.plos.org/plosone/s/materials-and-software-sharing#loc-sharing-code> and ensure that your code is shared in a way that follows best practice and facilitates reproducibility and reuse. | Thank you for your feedback. We acknowledge that this information was not yet made clear in the manuscript. We updated our ‘Data Availability Statement’ and now provide clear instruction on how the code can be fully accessed. | Change in the ‘Data Availability Statement’ (newly inserted text; please see l. 612)  Data for the creation of the FFTrees are publicly shared. Data are available here https://doi.org/10.17605/OSF.IO/KFRC2. The code underpinning our findings can be accessed through the R Package developed by Phillips et al. [42]. The latest developer version of FFTrees is available at http://www.github.com/ndphillips/FFTrees. The code and documentation presented here were created with the latest FFTree version 2.0.0. The specific formula used to create the FFTrees in this study is provided in the caption of the Supporting Information S 4 |
| Thank you for stating the following financial disclosure: [This work was supported by the Bundesinstitut für Sportwissenschaft (German Federal Institute for Sport Sciences) in 2021–2025 under Grant No. 081901/21-25 (English title: Individual performance development in elite sports by holistic and transdisciplinary process optimization). The data analyzed for the purposes of this study were part of a multidisciplinary large-scale data set that included multiple points of measurement and a cross-sectional and longitudinal perspective. The subset of data included in this study covered the cross-sectional data collected in the period 01/2022–12/2023.]. Please state what role the funders took in the study.  If the funders had no role, please state: """"The funders had no role in study design, data collection and analysis, decision to publish, or preparation of the manuscript."""" If this statement is not correct you must amend it as needed. Please include this amended Role of Funder statement in your cover letter; we will change the online submission form on your behalf. | Thank you for your inquiry. We apologize that this information was not adequately provided in the previous disclosure. We have now added to the statement that the funders had no role in the study (please find changes in the manuscript on the right). | Change in the financial disclosure  This work was supported by the Bundesinstitut für Sportwissenschaft (German Federal Institute for Sport Sciences) in 2021–2025 under Grant No. 081901/21-25 (English title: Individual performance development in elite sports by holistic and transdisciplinary process optimization). The funders had no role in study design, data collection and analysis, decision to publish, or preparation of the manuscript. The data analyzed for the purposes of this study were part of a multidisciplinary large-scale data set that included multiple points of measurement and a cross-sectional and longitudinal perspective. The subset of data included in this study covered the cross-sectional data collected in the period 01/2022–12/2023. |
| In the online submission form, you indicated that [The raw data supporting the conclusions of this article will be made available by the authors upon request, without undue reservation.]. All PLOS journals now require all data underlying the findings described in their manuscript to be freely available to other researchers, either 1. In a public repository, 2. Within the manuscript itself, or 3. Uploaded as supplementary information. This policy applies to all data except where public deposition would breach compliance with the protocol approved by your research ethics board. If your data cannot be made publicly available for ethical or legal reasons (e.g., public availability would compromise patient privacy), please explain your reasons on resubmission and your exemption request will be escalated for approval. | Thank you for your inquiry. We agree that this is essential for promoting Open Science. Of course, we will make the data available to you.  In that regard, we have made the dataset that went into creating and evaluating the FFTrees available through a public repository. This dataset encompasses data from all athletes who were considered for the creation of the FFTrees. However, for ethical reasons, we are unfortunately not able to provide the entire dataset containing competition data and the entire sample used for the PCA. These datasets contain sensitive information that could compromise patient privacy due to the broad spectrum of highly specific data.  We have updated the ‘Data Availability Statement’ to reflect these changes. | Change in the ‘Data Availability Statement’ (please see l. 612)  Data for the creation of the FFTrees are publicly shared. Data are available here <https://doi.org/10.17605/OSF.IO/KFRC2>. The code underpinning our findings can be accessed through the R Package developed by Phillips et al. [42]. The latest developer version of FFTrees is available at <http://www.github.com/ndphillips/FFTrees>. The code and documentation presented here were created with the latest FFTree version 2.0.0. The specific formula used to create the FFTrees in this study is provided in the caption of the Supporting Information S 4. |
| We are unable to open your Supporting Information file S4.1 trampoline.eps and S4.2 volleyball.eps. Please kindly revise as necessary and re-upload. | Thank you for drawing our attention to the problem. We apologize for the inconvenience. As a solution, we have changed the file format to PDF. We have re-uploaded the affected supplementary files and successfully tested them. | The problem did not require any changes in the manuscript. |

**Dear Reviewer #1**, we would like to sincerely thank you for your insightful and encouraging review of our manuscript. We greatly appreciate the time and effort you dedicated to carefully evaluating our paper. We are truly excited and grateful that you recognize the importance and value of the research topic we have addressed. Your positive assessment and summary affirm the relevance of our work and reinforced our enthusiasm for pursuing this research. We have carefully considered your comments as we finalized the revised manuscript.

| **Comments Reviewer #1** | **Answer** | **Change in the manuscript** |
| --- | --- | --- |
| The use of Fast-and-Frugal Trees (FFTs) in sports performance forecasting represents an interesting methodological advance that addresses the need for rapid, transparent, and efficient decision-making in contexts where time and clarity are critical, such as sports. Unlike complex statistical models or deep learning algorithms, FFTs offer a simplified decision structure that prioritizes speed and straightforward interpretation, which is especially valuable for coaches.  One of the study's key strengths is its ability to balance accuracy and usability. FFTs are easily understandable and applicable in real-world settings without requiring advanced software, which favors their implementation in sports clubs, youth national teams, and development programs. Furthermore, their explicit, hierarchical design facilitates communication between analysts and coaching staff. | Thank you for your feedback. We are grateful for your encouraging remarks about our methodological approach. Your acknowledgment of the importance of creating an accurate tool while keeping its usability in mind aligns closely with our motivation for selecting FFTrees. We aimed for fluid connection between data analysis, statistical approaches, and practical implications for coaches and practitioners. We are encouraged by your recognition of the potential for FFTrees to be adopted in diverse settings. We are glad that our methodology and considerations resonates with you. |  |
| However, it is important to recognize **certain limitations** inherent to the model. While FFTs perform well in binary decision settings or when speed is prioritized over complexity, they may have lower predictive capacity than more robust models (such as Random Forests or neural networks) in contexts with nonlinear data or many interactions between variables. | Thank you for your comment. We appreciate your feedback and have incorporated a paragraph to recognize the limitations.  We feel that the selection of FFTs in this specific case with a binary selection and a complex pattern of variables seems well justified. We fully acknowledged that other approaches might be informative and therefore,  we have rephrased the description of our results. Our findings show, in comparative testing FFTrees were found to be more effective than other models such as RF. This is consistent with the findings of other studies. | Change (please see l. 441)  Compared to alternative classification algorithms such as logistic regression, heuristic approaches have been demonstrated to achieve higher accuracy in predicting data in uncertain domains with small sample sizes [18]. Our findings align with this sentiment in that FFTrees outperformed alternative classification methods. Although the created FFTrees show slightly lower accuracy in testing compared to training data (S 5), this decline is to be expected. In contrast, overfitting is evident in the RF algorithm that attains optimal values in training data but cannot maintain this accuracy in testing data. This finding is consistent with prior research indicating that the RF algorithm is susceptible to overfitting, particularly when the training set is small [50]. Deeper models, such as artificial neural networks, require even more data. Empirical guidelines recommend a minimum of 10 to 20 observations per weight as necessary to ensure reliable generalization [51]. Additional analysis (i.e., ROC curves) demonstrates that in comparative testing in accordance with the second principle of Raab [14] defined as “test heuristics comparatively,” FFTrees exhibit a lower risk for overfitting and enhanced generalization.  Change (newly inserted text; please see l. 553)  The findings of this study have to be seen in light of certain limitations. For team sports, FFTrees may require position-specific trees to adequately represent the heterogeneous characteristics of different positions in volleyball and other team sports. Given the current sample size, implementing this approach is not yet feasible. Additionally, FFTrees exhibit instability across random partitions. A possible solution can be provided by increasing the sample size and expanding the data on which FFTrees are created. Numerous junior squad volleyball players were excluded due to a lack of individual performance records at their competition level. Generalization to these squad levels as well as to non-German athletes should be realized with caution. Hence, a comprehensive individualization approach is required that not only encompasses the diagnostics and interventions but also includes methods for performance development and competition diagnostics that differentiate individual contributions in team settings. |
| Furthermore, their performance can be **sensitive to the initial feature selection,** which requires methodological care in the design stage. | Thank you for your feedback.  We considered this as an important point that needed to be emphasized in the manuscript so that further studies on FFTrees can be guided by it.  To strengthen this, we have added your point for a well-thought-out design concept for the initial feature selection for FFTrees in the discussion. | Change (newly inserted text; please see l. 513)  Therefore, a sound methodological concept for feature selection in the initial design stage of FFTrees needs to be implemented. A comprehensive understanding of the requirements inherit to the sport can inform which variables should be incorporated into the FFTree creation process. The use of a PCA can provide statistical information on which areas of intervention exist and the diagnostic that best represent them. Additionally, we recommend cross-validating the created FFTrees with established research findings to ensure their plausibility. |
| This study constitutes a valuable contribution to the field of sports performance by demonstrating that it is possible to build agile and accessible predictive models without sacrificing their practical utility. The use of FFTs invites us to rethink how informed decisions are made in everyday sports contexts, highlighting that heuristic simplicity can be a virtue in complex environments, provided it is supported by rigorous empirical evidence. | Thank you for your generous comment. We are delighted that you recognize the value of our contribution to the field of sports performance. We appreciate your thoughtful reflection on the practical implications of our use of FFTrees. Your observation that, when grounded in rigorous empirical evidence, heuristic simplicity can be a valuable asset in complex environments, truly resonates with the core message we hoped to convey. |  |

**Dear Reviewer #2,** we would like to thank you sincerely for your thorough and detailed review of our manuscript. We are grateful for the careful attention and effort you invested in reviewing our paper. Your thoughtful and detailed feedback, including the helpful examples, and practical suggestions for improvement, has been incredibly valuable to us. Your constructive feedback has inspired us to refine and strengthen the manuscript further. We carefully considered all of your recommendations while revising the manuscript.

| **Comments Reviewer #2** | **Answer** | **Changes in the manuscript** |
| --- | --- | --- |
| **Abstract** |  |  |
| The **structure** could be improved for clarity. Consider explicitly segmenting the abstract into: Objective, Methods, Results, Conclusion... | Thank you for your feedback. We totally agree. As you suggested, we have now formulated a clearer objective by changing the structure and introducing clearer segments in the abstract. In addition, we have added phrases introducing the different segments (please find changes in the manuscript on the right).  We hope this will further improves readability and clarity of the abstract. | Change (newly inserted text; please see abstract l. 24)  The objective of this study was to examine the applicability of FFTrees to inform intervention decisions in elite athletes. We aimed to create FFTrees and evaluate their ability to determine individually beneficial interventions.  Change (please see abstract l. 28)  First, we used principal component analysis to identify components representing types of interventions across sports.  Change (please see abstract l. 31):  As a result, the PCA identified six cues. Two sport-specific FFTrees were created using these six cues. One FFTree was created for trampoline with four cues (relative grip strength, motor cost, motor inhibition, visual selective attention) and 90% correct predictions. The other FFTree was created for volleyball with four cues (motor inhibition, motor cost, countermovement jump, Y-Balance Test) and 75% correct predictions.  Change (please see abstract l. 36)  To conclude, the high accuracy confirms that FFTrees enable data-based decisions for interventions based on sport-specific demands and the preferences of coaches.  Change (please see abstract l. 41)  In the future, we advocate that team sports develop of position-specific FFTrees. In conclusion, FFTrees empower decision-makers by efficiently identifying athletes’ adaptation potentials. |
| **Clarity of FFTrees**: As this might be unfamiliar to some readers, consider briefly defining what FFTrees are in one sentence near the start. | Thank you for your helpful feedback. We agree that there might remain uncertainties for the reader after the abstract. We have now added a sentence to the abstract to give a definition for FFTrees. | Change (newly inserted text; please see abstract l. 23)  FFTrees are binary decision trees that can make decisions based on only one reason. |
| **Introduction** |  |  |
| **Objective not clearly stated**: Lines 101-104: The objective appears but is somewhat embedded. You may consider adding a clear and concise sentence like: “The aim of this study is to develop and evaluate the predictive performance of sport-specific FFTrees to inform personalised training interventions in elite athletes.” | Thank you for suggesting that an explicit objective would improve our manuscript. We agree that the objective was somewhat lost in the paragraph. We have now revised the respective paragraph for clarification. To enhance clarity, we restructured the section and now used the word ‘aim’ more selectively. | Change (newly inserted text; please see l.106)  Therefore, our study will deliver a proof of concept by developing FFTrees and evaluating their ability to determine individualized needs for interventions from multifaceted diagnostics in elite sports. Because different sports feature different specific characteristics and requirements, the decision for an intervention should be evaluated for each athlete and for each sport disciplines. In this article, we consider differences between individual and team sports to be most fundamental, and we therefore validated sport-specific FFTrees for trampoline and volleyball to represent these two fundamental categories. In summary, the study overall aims to create sport-specific FFTrees as an intuitive decision tool to inform personalized training interventions in elite athletes.  More specifically, to achieve this aim, we showcase the reduction of complexity resulting from reducing multidisciplinary diagnostics to a few components in order to derive types of interventions facilitating the selection of variables used as cues for FFTrees. |
| **Theory clarification**: Lines 31-40: You refer to theoretical models but do not fully explain how these support the rationale for FFTrees. Consider integrating the constructivist or biopsychosocial lens more explicitly to frame why FFTrees are an appropriate tool for this context. Suggestion for structure: You might consider adding subheadings or clearly defined paragraphs separating: Context and problem (elite sports complexity); Existing gaps in intervention selection; Introduction to FFTrees as a methodological response | Thank you for your feedback.  We have incorporated information to now provide a better understanding on why FFTrees might be an appropriate tool. | Change (newly inserted text, please see l. 73)  This is in contrast to the assumptions of unbounded rationality, in which knowledge of all probabilities, consequences, and alternatives is assumed [12]. The assumptions of unbounded rationality are not met. FFTrees are an appropriate tool for handling the uncertain relationships between results obtained from multidisciplinary diagnostics and athletes’ individual performance development. Given these circumstances, heuristics can maintain predictive accuracy while simplifying decision making by employing the less-is-more effect [13,14].  Change (newly inserted text, please see l.85)  The identified sequence of cues represents the prioritization of variables for the decision according to their validity, thereby giving priority to the cue with the highest probability for a prediction [13]. The decision is terminated via a cue that allows a classification defined by the cut-off value through the stop rule, ignoring all subsequent lower-level cues [16]. FFTrees are therefore considered frugal [13]. The decision rule informs, in our case, which type of intervention is most important to consider [9]. FFTrees have the capability of facilitating prioritization among the various multidisciplinary areas of intervention. |
| **Methods** |  |  |
| **Cue Selection Explanation**: Lines 210-225: The PCA is well described but consider clarifying why each variable was selected as a cue. For example, in line 299-301, you could briefly mention the rationale for replacing information-processing speed with visual selective attention in trampoline | Thank you for this hint. We agree that this enhances clarity in this section.  As you proposed, we changed this in the manuscript and have now added information on how cues were specifically selected or exchanged for the sport specific FFTree. | Change (newly inserted text, please see l. 311)  The rationale behind this was the consideration of variables with the highest loading within a component as well as the fewest missing values. |
| **Clarify Dichotomisation Criteria**:  Lines 234-245: Please make the thresholding process more transparent. For instance:  How was the “75th percentile” decided?  Were sex and age considered in the dichotomisation? | Thank you for your helpful feedback. Sorry that this process lacked clarity in our first draft.  We examined international qualifying competition results and compared them with our athletes’ competition data. We found that athletes who placed 50th in international competitions achieved a competition score reflective of the 75th percentile in our athletes  Sex and age were considered to the extent that competition data were only held and judged exclusively within gender and age groups.  To increase transparency of this section, we now provide more detailed information on how the 75th percentile was decided as well as on how sex and age were considered for the thresholds (please find changes on the right).  In addition, we emphasized the section in the discussion that elaborates on the decision for the specific threshold. (please see l. 557)  We hope that this increases overall comprehension of the chosen thresholds. | Change (please see l. 241)  For both selected competitions—the 37th FIG Trampoline Gymnastics World Championships in Birmingham and the Qualifying OG for the FIG World Cup 2024—the 50th place in the qualification round was used as a reference value to guide the selection of the threshold. The volleyball threshold was aligned with the trampoline threshold for comparison reasons. To accommodate for differences in competition performance regarding the playing positions in team sports, partitioning was position-specific. The thresholds were established separately from considerations of sex and age, because competitions are only held and judged exclusively within gender and age groups.  Change (please see l. 547)  The 75th percentile was selected as a conservative threshold. This threshold provides enough space for detecting needs for interventions and balancing sensitivity and specificity. |
| **Reproducibility**:  Lines 272-273: You may consider adding the R script or relevant code as supplementary material or sharing a GitHub link, in line with open science practices. | Thank you for pointing this out. We agree that in line with open science practices code sharing would provide additional value. Therefore, we updated our Data Availability Statement and reference to the GitHub link, and the publication associated with the used code. The specific formulas used to generate the FFTrees are available in the caption of the Supporting Information S 4 | Change (newly inserted text; please see l. 613)  The code underpinning our findings can be accessed through the R Package developed by Phillips et al. [42]. The latest developer version of FFTrees is available at http://www.github.com/ndphillips/FFTrees. The code and documentation presented here were created with the latest FFTree version 2.0.0. The specific formula used to create the FFTrees in this study is provided in the caption of the Supporting Information S 4. |
| **Results** |  |  |
| Table 3.1 (line 293): It would be helpful to add a brief sentence interpreting **the practical relevance of each component**, what does “motor cost” imply, for instance? | Thank you for your suggestion. As you proposed, we have added the practical relevance of each component to now provide a better understanding and show the relevance for sports practice. | Change (please see l. 295)  The fifth component consisted of motor cost, which is indicative for the dual task performance. Athletes with low motor cost can perform motor and visual cognitive tasks simultaneously without experiencing high interference between the tasks that produce motor task decrements. Motor inhibition loaded on the sixth component, representing the ability of athletes to interrupt and stop a planned action after a visual stimulus indicating to stop the movement. |
| Figures 3.1 and 3.2: The decision rules could be better **explained in-text**. For example, in line 327-328, you might add: ⎫“This threshold suggests that athletes with grip strength z-scores > -0.12 are classified as needing intervention, reflecting possible strength deficiencies.” | Thank you for your feedback. We appreciate your opinion to provide a better understanding on what and how the decision process can look like. Therefore, as you suggested, we have incorporated an in-text decision process for the trampoline and volleyball FFTree as suggested. Additionally, we included an illustrative decision-making process for an exemplary athlete A in the discussion. | Change (please see l. 333)  The threshold suggests that once athletes’ relative grip strength *z*-values were above -0.12, they can be classified as needing intervention. All following cues are ignored. Scoring below this threshold will lead to the assessment of the motor costs. An individual motor cost *z* value less than or equal -0.49 classifies athletes as not being in need for an intervention. Values above 0.49 would indicate deficiencies in dual task performance. However, a definite decision will only be made in subsequent cues such as motor inhibition.  Change (newly inserted text; please see l.357)  Athletes with motor costs less or equal to a z value of -0.20 are not in need for an intervention. All athletes having values above -0.20 could show a decrement in dual task performance and subsequent cues must be considered. The threshold for CMJ suggests that athletes with a z value below -0.47 are classified as needing intervention. Similarly, the threshold z value of -0.63 for the YBT cue classifies athletes as requiring mobility interventions.  Change (please see l. 503)  Consider the volleyball player A, who exhibits high motor inhibition and low motor costs. However, his CMJ performance is below the threshold. In this scenario, the FFTree suggests the implementation of strength interventions within in the athlete’s training regime to enhance performance. The performance of athlete A in the YBT would not affect the decision and can be ignored. The intervention with the highest priority for the athlete is identified. The FFTree can be used again to find additional intervention needs if capacities for further interventions are available. |
| **Missing data handling**: Line 256-258: Please state how many cases were excluded due to missing data, or clarify if imputation was considered. | Thank you for your comment. We agree that this was not clear in our first draft.  As you suggested, we have rephrased the information in the methods and now provide a clearer description on how missing data was handled. We also enhanced the results section, which specified the number of cases excluded due to missing data. | Change (please see l. 259)  Athletes with missing values in a particular cue were ignored when analyzing that particular cue, but were included in all unaffected cues. No imputation for missing data was performed, given that the other values remained unaffected and the sample size could be retained. Consequently, there was no risk of bias associated with the missing data.  Change (please see l. 373)  In the trampoline FFTree, only one cue contained a missing value from one athlete in motor cost. The athlete was therefore excluded for the analysis of the motor cost cue. In the volleyball FFTree, a quota of 5.6% missing values could be found in CMJ, hedonic balance, motor cost, motor inhibition, and information-processing speed. In the analysis of the respective cues, the missing values were omitted from the analysis. |
| **Discussion** |  |  |
| **Theoretical anchoring**: Lines 382-390: While you reference the multidisciplinary and heuristic value of FFTrees, it would strengthen the paper to briefly connect to models such as constructivist decision-making or ecological dynamics, especially since you touch on bounded rationality. This would deepen the theoretical contribution. | Thank you for your comment. We agree further integration of underlying theoretical consideration would improve understanding and reasoning. As you proposed, we have inserted a paragraph on theoretical contributions to the manuscript and now provide additional references. | Change (newly inserted text; please see l. 411)  FFTrees are suitable tools in elite sports as we need to consider time and training constraints that require us to make decision on which athletes to prioritize for specific interventions. FFTrees rely on the concept of bounded rationality. This is based on Herbert Simon’s [46] view addressing the limitations of our mind and the structure of the environment in which decisions unfold. Such bounded rationality perspective is not specific to FFTrees as evident for instance in Brunswik’s [47] work or ecological approaches [48]. A difference between ecological approaches and heuristics, however, is that while ecological approaches consider person and environment as one system, heuristic programs consider them interacting components (see Raab & Araujo [49] for a discussion of differences in sports). |
| **Practical implications**: Lines 445-455: Consider adding an example of how a coach could use a FFTree in a training context. For example: “A coach reviewing FFTree output could prioritise strength-based interventions if the tree identifies grip strength or CMJ as first-level cues.” | Thank you for your suggestion. As you proposed, we have incorporated an additional paragraph adding use cases.  We agree that this highlights the practical implications of FFTrees. | Change (newly inserted text; please see l. 496)  In practice, a coach reviewing the FFTree can gather information regarding which areas for intervention are most important for potential performance improvements. This can be seen in the order of the cues within the FFTree. The higher the cue, the more important it is. For instance, strength-based interventions merit high priority if the FFTree identifies relative grip strength or CMJ as a first-level cue. Furthermore, the cut-off values offer norm values appropriate for elite athletes. The primary benefit coaches derive from FFTrees is the ability to directly assess and categorize diagnostic information from athletes, thereby informing decisions regarding training regimes. |
| **Limitations**: While limitations are mentioned (Lines 491-494), it would benefit from a clearer subsection or paragraph that includes:  Small sample sizes for team sports  Model instability across random partitions  Generalisation limitations to youth or non-German athletes | Thank you for your comment. We appreciate your intake and have introduced a paragraph summarizing the limitations of our study. Additionally, we added considerations on the limitations on generalization for non-German athletes were added. | Change (newly inserted text; please see l. 553)  The findings of this study have to be seen in light of certain limitations. For team sports, FFTrees may require position-specific trees to adequately represent the heterogeneous characteristics of different positions in volleyball and other team sports. Given the current sample size, implementing this approach is not yet feasible. Additionally, FFTrees exhibit instability across random partitions. A possible solution could be increasing sample size and expanding the data on which FFTrees are created on. Numerous junior squad volleyball players were excluded due to a lack of individual performance records at their competition level. Generalization to these squad levels as well as to non-German athletes should be realized with caution. Hence, a comprehensive individualization approach is required that not only encompasses the diagnostics and interventions but also includes methods on performance development and competition diagnostics that differentiate individual contributions in team settings. |
| **Conclusion** |  |  |
| Line 511 onward: The conclusion is strong but consider **softening claims** slightly to reflect the proof-of-concept nature of the study. For example: “These preliminary findings suggest that FFTrees may offer a practical, transparent method for guiding individualised training decisions, though further research with larger samples is recommended.” | Thank you for your comment. We appreciate your feedback to better reflect the nature of our study. As you suggested, we have adjusted the conclusion by softening the claims. We hope this fits the proof of concept nature of the study better. | Change (please see l. 590)  These findings suggest that FFTrees serve as a practical and efficient decision-making tool in sports science, offering an approach to unlock athletes’ true adaptation potential.  Change (please see l. 605)  Expanded research with larger sample sizes could produce more stable FFTrees and lead to position-appropriate intervention decision. |
| **Language & Style** |  |  |
| Orthographic errors: Line 101: “and whether they can be validated…” is incomplete or has a clause error. Line 249, 304, and several others: Consider reviewing sentence punctuation. Several sentences are long and lack commas, which makes them harder to follow, this may stem from AI-assisted generation. Recommendation: A thorough proofreading by a native English speaker or professional editor would improve clarity and flow and meet the PLOS ONE criterion for “standard English.” | Thank you for your feedback. Before we submitted, a native English speaker originally edited the manuscript. However, you are absolutely right. We apologize that the English in our manuscript contained orthographic errors and did not meet the criteria for standard English. We have now revised the entire manuscript thoroughly and corrected the orthographic errors. In addition, we shortened some longer sentences in the discussion (please see the ones you explicitly mentioned and some other changes on the right, for all adjustment please refer to the manuscript with tracked changes). | Change (please see l.103)  There is still a research gap in sports regarding how FFTrees can be applied to individual performance development and whether they can be validated through comparisons to alternative methodologies in intervention selection.  Change( previously l. 249; please see l. 255)  Part of the data, in our case 60%, was used to train the FFTree algorithm. The FFTrees were tested on the remaining 40% of the data to estimate predictive performance.  Change (previously l.304; please see l. 316)  For comparison with international qualifying competitions, female athletes reached 49.40 and 50.86 points, and male athletes 54.71 and 53.96 points. These results correspond to the 75th percentile in the in:prove population (total score_female_ = 48.43; total score_male_ = 51.04).  Change (please see l. 404)  One FFTree was created for trampoline (individual sport) and one for volleyball (team sport) based on diagnostics of elite athletes and current competition performance. This ensures real-world testing in accordance with the fifth principle of Raab [14] defined as “test heuristics in the real world.” The creation of FFTrees incorporates biopsychosocial determinants*,* reflected in the selection of multidisciplinary cues [7]. FFTrees bridge the multidisciplinary understanding of expertise with individualized intervention selection.  Change (please see l. 428)  However, decisions for no intervention (e.g., motor cost) do not provide clear identification of adaptation potential. These decisions require the assessment of subsequent cues such as CMJ or YBT.  Change (please see l.435)  Dataset partitioning makes it possible to assess testing data in order to detect overfitting. By evaluating how well FFTrees predict data, we follow the fourth principle of Raab [14] defined as “examine how well models of heuristics predict new data.” |

**Dear Reviewer #3,** we are sincerely grateful for your thoughtful evaluation of our manuscript. We deeply appreciate the time and expertise you dedicated to review our paper. Your insightful feedback and practical suggestions for improvement, have been incredibly valuable to us. Your observations have played a significant role in strengthening our manuscript further. We carefully considered all your recommendations while revising the manuscript.

| **Comments Reviewer #3** | **Answers** | **Changes in the Manuscript** |
| --- | --- | --- |
| The **title** is somewhat **generic**. Given the strong applied dimension of this study, I suggest emphasizing the performance enhancement aspect in the title to increase its attractiveness and specificity. | Thank you for your feedback. We agree that our chosen title is rather generic. Therefore, we have adjusted the title to your feedback to be more precise (please see to your right). | Before  Fast-and-frugal trees: A decision tool for elite sports  Change (please see title page l.1)  How fast-and-frugal trees can inform diagnostic and intervention decisions for enhancing elite athlete performance. |
| Please consider rewriting the final lines of the abstract to emphasize the **contribution to performance science and coaching practice** more clearly. | Thank you for your recommendation. We have now added a sentence to promote a clearer visualization on the contribution. | Change (newly inserted text; please see abstract l. 37)  We argue that FFTrees are beneficial in projects collecting multidisciplinary variables for personalizing interventions in elite athletes. Coaching practice benefits from using FFTrees by providing reference values when an intervention could enhance performance. In the future, we advocate that team sports develop of position-specific FFTrees. In conclusion, FFTrees empower decision-makers by efficiently identifying athletes’ adaptation potentials |
| Although the methodological approach is sound, the sample sizes (n = 27 for trampoline, n = 53 for volleyball) are relatively small. This particularly limits generalizability for the trampoline model and should be addressed more **explicitly in the limitations section**. | Thank you for your suggestion. We agree that the topic of small sample sizes needs for further clarification and can be better incorporated in the limitations. Therefore, we have added a limitation paragraph and mentioned the small sample size more explicitly. In addition, we have provided a possible solution on how future research could handle the issue of small sample sizes.  Additionally, through emphasis and discussion of the performance between the FFTrees for individual and team sport (see l. 459) we highlighted that despite the small sample sizes in trampoline the predictive performance was as good and even better compared to volleyball. This indicates that the trampoline FFTree aside from the instability in general can be a good decision tool and provide acceptable generalization. In elite sports we encounter the problem of limited sample size due to the selective nature of the environment. Hence, we need statistical solution that can provide acceptable results under these circumstances. | Change (newly inserted text; please see l. 523)  Previously generated FFTrees [24,42,58] used considerably larger samples than the present study. One potential solution to the inevitable small sample sizes in elite sports might be the aggregation of data from multiple years, thereby creating a more substantial database. This would provide an increasing cohort for generating and refining FFTrees.  Change (newly inserted text; please see l. 533)  The findings of this study have to be seen in light of certain limitations. For team sports, FFTrees may require position-specific trees to adequately represent the heterogeneous characteristics of different positions in volleyball and other team sports. Given the current sample size, implementing this approach is not yet feasible. Additionally, FFTrees exhibit instability across random partitions. A possible solution could be increasing sample size and expanding the data on which FFTrees are created on. |
| What specific **challenges** did the research team face while athletes completed questionnaires or diagnostic tests, given the constraints of elite training environments? Clarifying this in the discussion or limitations section may be insightful. | Thank you for highlighting the importance of addressing specific challenges we encountered. We appreciate your suggestion as it draws attention to the critical aspect, we needed to consider while creating our FFTrees. We have emphasized the challenges we faced while capturing diagnostic data. | Change (please see l. 535)  Managing missing values is an essential challenge in elite sports. Injuries and other health-related concerns can limit participation in diagnostic procedure. |
| Clearly identify the cognitive cues used for each sport to help readers better **understand sport-specific adaptations of the FFTrees**. | Thank you for your feedback. We agree that the explanation of how the sport-specific adaptation were made was somewhat vague. In response, we have adapted the section on sport-specific adaptation to provide a clearer understanding of how the different cues were selected. | Change (newly inserted text, please see l. 311)  The rationale behind this was the consideration of variables with the highest loading within a component as well as the fewest missing values. |
| Consider simplifying or **contextualizing the key outputs** (e.g., cut-off values, cue combinations) in a way that is easier for coaches at various levels to interpret and apply. | Thank you for your suggestion. We agree additional context would be beneficial for comprehension. We have added a section that give context on how different outputs can provide information for coaches. | Change (newly inserted text; please see l. 496)  In practice, a coach reviewing the FFTree can gather information regarding which areas for intervention are most important for potential performance improvements, as evidenced by the first cue. For instance, strength-based interventions merit high priority if the FFTrees identifies relative grip strength or CMJ as a first-level cue. Furthermore, the cut-off values offer norm values appropriate for elite athletes. The primary benefit coaches derive from FFTrees is the ability to directly assess and categorize diagnostic information from athletes, thereby informing decisions regarding training regimes. |
| It would enhance the practical relevance of the study to provide concrete**, real-world examples** of how each cue can be implemented in athlete monitoring or decision-making. | Thank you for your comment. We appreciate your suggestion on how to enhance the relevance of our study. We added information on the practical relevance of each component in the results. We have provided additional examples on how the cues can be implemented. | Change (newly inserted text, please see l. 289)  The first component contained five variables (CMJ, DJ, sprint, tapping, and relative grip strength) belonging to the category of motor-performance diagnostics (focusing on power and speed diagnostics). Component two consisted of five variables from the psychosociological diagnostics (burnout, social support, group cohesion, hedonic balance, and general life satisfaction). Information-processing speed and visual selective attention, both cognitive diagnostics, loaded on the third component. Mobility diagnostics YBT and KtW were assigned to component four. The fifth component consisted of motor cost, which is indicative of dual task performance. Athletes with low motor cost can perform motor and visual cognitive tasks simultaneously without experiencing high interference between the tasks that produce motor task decrements. Motor inhibition loaded on the sixth component, representing the ability of athletes to interrupt and stop a planned action.  Change (newly inserted text; please see l. 498)  For instance, strength-based interventions merit high priority if the FFTrees identifies relative grip strength or CMJ as a first-level cue.  Change (newly inserted text; please see l. 503)  Consider the volleyball player A, who exhibits high motor inhibition and low motor costs. However, his CMJ performance is below the threshold. In this scenario, the FFTree suggests the implementation of strength interventions within in the athlete’s training regime to enhance performance. The performance of athlete A in the YBT would not affect the decision and can be ignored. The intervention with the highest priority for the athlete is identified. The FFTree can be used again to find additional intervention needs if capacities for further interventions are available. |
| Several sentences in the discussion are long and dense. **Revising into shorter, clearer statements** would improve readability. | Thank you for your feedback. We agree that some sentences were long which could hinder smooth comprehension. We have adapted and revised several sentences to break down complex sentences into shorter, clearer statements (please see some changes on the right, for all adjustment please refer to the manuscript with tracked changes). We hope this improves readability. | Change (please see l. 404)  One FFTree was created for trampoline (individual sport) and one for volleyball (team sport) based on diagnostics of elite athletes and current competition performance. This ensures real-world testing in accordance with the fifth principle of Raab [14] defined as “test heuristics in the real world.” The creation of FFTrees incorporates biopsychosocial determinants*,* reflected in the selection of multidisciplinary cues [7]. FFTrees bridge the multidisciplinary understanding of expertise with individualized intervention selection.  Change (please see l. 428)  However, decisions for no intervention (e.g., motor cost) do not provide clear identification of adaptation potential. These decisions require the assessment of subsequent cues such as CMJ or YBT.  Change (please see l.435)  Dataset partitioning makes it possible to assess testing data in order to detect overfitting. By evaluating how well FFTrees predict data, we follow the fourth principle of Raab [14] defined as “examine how well models of heuristics predict new data.” |
| Please elaborate more on the **difference in FFTree performance** between individual (trampoline) and team sports (volleyball). This is an important insight for readers. | Thank you for your comment. We appreciate your suggestion to offer more insights on the differences. Your acknowledgement of the importance of this matter motivated us to provide additional information. We have added a section addressing the differences between individual and team sports. Additionally, we have clarified the difference in a separate limitation paragraph. | Change (newly inserted text; please see l. 456)  FFTrees reveal higher predictive accuracy for trampoline (73 ± 14%, max. 90%) than for volleyball (58 ± 11%, max. 75%.) despite the smaller sample size in the former. The disparity in FFTree performance between individual and team sports indicates that the FFTree for trampoline can more effectively capture the structure of the performance determinants. In volleyball, however, the FFTree is not quite capable of correctly classifying all volleyball players using the same set of decision rules. This discrepancy may be due to the more complex and uncertain structure of team sports such as many different games systems (i.e., tactics), and player positions that differ in their requirements and the respective athlete types [52–54]. Unlike individual sports, where every athlete is confronted with the same athletic requirements and performance goals, team sports are a collection of different player positions with different tasks specific for their position.  Change (newly inserted text; please see l. 553)  The findings of this study have to be seen in light of certain limitations. For team sports, FFTrees may require position-specific trees to adequately represent the heterogeneous characteristics of different positions in volleyball and other team sports. Given the current sample size, implementing this approach is not yet feasible. |
| Correct the term "instable FFTrees" to "**unstable** FFTrees" for better clarity and technical accuracy. | Thank you for your suggestion. We agree that unstable is a better wording for the phenomenon and have changed it. | Change (please see l. 511)  In the following, this phenomenon is referred to as “unstable” FFTrees |
| If possible, discuss the **transferability** of this approach to other sports or organizational settings to broaden its application. | Thank you for your feedback. We have added information on how this approach can be transferred to other sports. We hope this strengthens the outlook for future studies. | Change (newly inserted text; please see l. 575)  Previous research has demonstrated the versatility of applications for FFTrees [18–24]. Additionally, Raab and Gigerenzer [9] have proposed FFTrees as a helpful approach to performance science. Consistent with this, our study has demonstrated the efficacy of FFTrees as a decision-making tool in elite sports. In order to expand upon our research and recent findings, we advocate the implementation of sport-specific and position-specific FFTrees in additional sports disciplines. The establishment of a transparent protocol for the selection of interventions tailored to the needs of athletes in various sports would be promoted by this. Simultaneously, it would provide insights into the requirement profiles and prioritization of multidisciplinary diagnostics for the respective sport. |
